# Complex dissemination of the diversified *mcr-1*-harbouring plasmids in *Escherichia coli* of different sequence types

**DOI:** 10.18632/oncotarget.12621

**Published:** 2016-10-12

**Authors:** Qingjing Wang, Zhencui Li, Jingxia Lin, Xiuna Wang, Xianbo Deng, Youjun Feng

**Affiliations:** ^1^ Department of Medical Microbiology and Parasitology, Zhejiang University School of Medicine, Hangzhou, Zhejiang, China; ^2^ College of Veterinary Medicine, South China Agricultural University, Guangzhou, Guangdong, China; ^3^ Fujian Agriculture and Forestry University, Fuzhou, Fujian, China

**Keywords:** MCR-1, diversity, sequence type (ST), Immunology and Microbiology Section, Immune response, Immunity

## Abstract

The emergence of the mobilized colistin resistance gene, representing a novel mechanism for bacterial drug resistance, challenges the last resort against the severe infections by Gram-negative bacteria with multi-drug resistances. Very recently, we showed the diversity in the *mcr-1*-carrying plasmid reservoirs from the gut microbiota. Here, we reported that a similar but more complex scenario is present in the healthy swine populations, Southern China, 2016. Amongst the 1026 pieces of *Escherichia coli* isolates from 3 different pig farms, 302 *E. coli* isolates were determined to be positive for the *mcr-1* gene (30%, 302/1026). Multi-locus sequence typing assigned no less than 11 kinds of sequence types including one novel Sequence Type to these *mcr-1*-positive strains. PCR analyses combined with the direct DNA sequencing revealed unexpected complexity of the *mcr-1*-harbouring plasmids whose backbones are at least grouped into 6 types four of which are new. Transcriptional analyses showed that the *mcr-1* promoter of different origins exhibits similar activity. It seems likely that complex dissemination of the diversified *mcr-1*-bearing plasmids occurs amongst the various ST *E. coli* inhabiting the healthy swine populations, in Southern China.

## INTRODUCTION

Antibiotic resistance (referred to bacterial drug resistance here) has already developed into a leading problem in global public health [1]. The notorious New Delhi β-lactamase 1 (NDM-1)-producing super-bugs that was initially found in India, Pakistan and the UK [2] had ever pushed us on the cusp of post-antibiotics era. The prevalence of NDM-1 in the Gram-negative Enterobactereae including *E. coli* and *Klebsiella pneumoniae* (*K. pneumoniae*) confers the robust resistance to carbapenems and expanded-spectrum (or third-generation) cephalosporins, the two extensively-used antibiotics for treatments of multidrug-resistant bacteria [3, 4]. The polymyxin E (colistin), a family of cationic polypeptide antibiotics, might represent the last line of defense against lethal infections by Gram-negative pathogens with pan-drug resistance [1]. However, it seems likely that this ultimate line of refuge antibiotics (polymyxin), has been challenged by the emergence of colistin resistance mechanisms [1, 5]. Among them, one refers to the chromosome-encoded machinery (two sets of bacterial two-component systems [*pmrAB* [6] and *phoPQ* [7]) and the regulator *mgrB* [7] are implicated in *K. pneumoniae* [7], and the other denotes the plasmid-mobilized colistin resistance (MCR-1) in certain species of *Enterobacteriaceae*, e.g.: *E. coli* [8]. The above two mechanisms are responsible for chemical modification of the lipid A on bacterial LPS, which consequently resulting in the reduced affinity for the polymyxin [8].

The *mcr-1* gene defines a newly-emerging mechanism for plasmid-mediated transferable colistin resistance [8]. The *mcr-1* protein product, MCR-1, is predicted to be an integral membrane protein with the catalytic activity of phosphoethanolamine transferases [9]. The MCR-1 enzyme modifies the chemical structure of lipid A moiety on bacterial LPS by the addition of phosphoethanolamine, which in turn reduces the binding affinity to colistin (*i.e.*, producing the colistin resistance) [8, 9]. Since the first discovery of MCR-1 from Southern China, in the late of 2015, the new colistin resistance gene has spread to 5 of 7 continents [10]. A retrospective study by Shen *et al*. [11] reported that the *mcr-1* gene is detected in three chicken *E. coli* isolates derived from the 1980s, when colisitin first started to be used in food-producing animals in China. It suggested that the emergence of the MCR-1 is much earlier than we anticipated [11]. To the best of our knowledge, no less than six species of Enterobacteriaceae (*E. coli* [9, 12–14], *Enterobacter aerogenes* [15], *Enterobacter cloacae* [15], *K. pneumonia* [16–18], *Salmonella enterica* [19–21], and *Shigella somnei* [22] are recipient hosts for the *mcr-1*-harbouring plasmids [10]. In particular, the MCR-1-producing plasmids display unexpected diversity, indicating the complexity of the MCR-1 dissemination mechanism [9]. The whole genome sequences of diversified *mcr-1*-carrying plasmids allowed us to better understand the mechanisms for the origin, evolution, transfer and dissemination of the *mcr-1* colistin resistance [13, 23–25].

Given the fact that 1) Guangdong province of China is the first place where the *mcr-1* gene was discovered [8]; 2) We observed that unexpected diversity in the *mcr-1*-harbouring plasmid reservoirs is present in the gut microbiota from the diarrhea patients in Shenzhen city localized in the same province Guangdong [9]; 3) Guangdong is one of the largest province for pig production in China, we therefore attempted to address the possibility whether complex dissemination of the MCR-1 colistin resistance by the diversified *mcr-1*-bearing plasmids is widespread in *E. coli* with different sequence types from healthy pig populations. In this paper, we report that this is the case.

## RESULTS AND DISCUSSION

### Occurrence of the *mcr-1*-positive *E. coli* isolates

**Figure 1:**
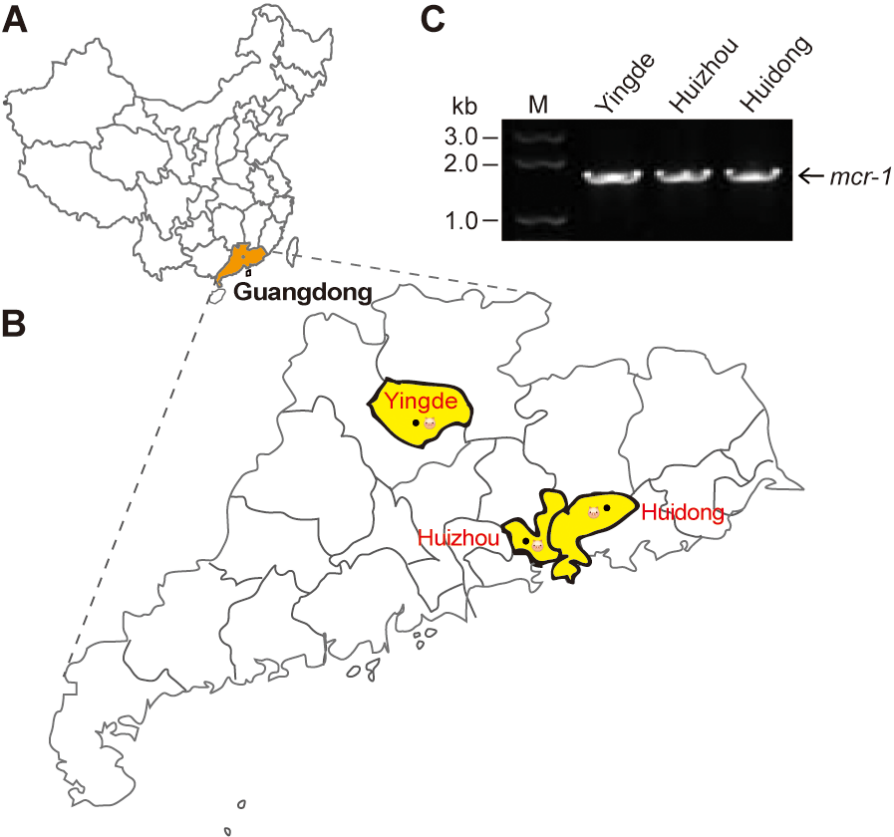
Distribution of the *mcr-1* colistin resistance gene in Guangdong province **A.** Location of Guangdong Province in China. Guangdong province is highlighted in orange. **B.** Locations of *mcr-1*-positive isolates from the swine gut microbiota in Guangdong Province. Locations are highlighted in yellow. **C.** The *mcr-1*-based screening of the isolates from the swine gut microbiota. The map is modified using adobe illustrator

In total, the bacterial samples were from three cities (and/or counties) of Guangdong Province (Figure 1A), which correspond to Yingde City, Huizhou City and Huidong County, respectively (Figure 1B). Each city hosted numbers of pig farms. In principle, nasal fluid and fecal samples were collected from piglets, fattening pigs and sows. The enteric bacteria were selectively screened on the MacConkey Agar plates, and the resultant single colonies were further subjected to the propagation in the liquid LB media (not shown). Subsequently, we conducted PCR screen for the presence of the *mcr-1* gene amongst the bacterial species. As expected, the *mcr-1*-positive isolates were found in the above three cities/counties (Figure 1C). Direct DNA sequencing results revealed that the *mcr-1* gene from hundreds of bacterial isolates is in 100% identity. Unlike the *mcr-1.2*, a variant (Q3L) of the *mcr-1* gene [16], we indeed failed to observe any alleic variants. 16S rDNA-based phylogenetic analyses proved that all the *mcr-1*-positive isolates are *E. coli* (not shown). As a result, 302 of 1026 *E. coli* isolates from the 3 distant pig farms were confirmed to be positive for the *mcr-1* gene in our trials. It suggested that the average positive rate of *mcr-1* is around 30% in these pig-producing places. Also, 15 representative *mcr-1*-positive clinical isolates were applied for further functional tests using the LBA plates supplemented with colistin at various levels. Consistent with our recent observation with the human clinical isolates that produce MCR-1 [9], they consistently exhibited the appreciable level of colistin resistance in that the minimum inhibitory concentration (MIC) is up to 32 mg/L (not shown).

**Table 1:** Strains used in this study

Strains or plasmids	Relevant characteristics	Origin
Strains		
MC1061	Wild type of *E. coli* K-12, Δ*lac*	[32]
DH5a (λ-pir)	Δ*lac* host for pAH125 and its derivatives	[32]
GD97	*E. coli* carrying pGD97, the *mcr-1*-harbouring plasmid	[12]
WH13	*E. coli* carrying pWH13, the *mcr-1*-harbouring plasmid	[12]
E15017	*E. coli* carrying pE15017, the *mcr-1*-harbouring plasmid	[9]
A31-12	*E. coli* carrying the *mcr-1*-harbouring plasmid pA31-12	[25]
MG1655	The wild type K-12 strain of *E. coli*	Lab stock
FYJ795	MG1655 carrying pBAD24::*mcr-1*	[9]
FYJ796	MG1655 carrying pBAD24	[9]
FYJ158	DH5α (λ-*pir*) carrying pAH-P*fadD*	[31]
FYJ846	DH5α (λ-*pir*) carrying pAH-P*mcr-1*(pGD97)	This work
FYJ847	MC1061 with P*mcr-1*_pWH09-*lacZ* transcriptional fusion at the chromosomal attB λ site	This work
FYJ848	DH5α (λ-*pir*) carrying pAH125-P*mcr-1*(pWH13)	This work
FYJ849	MC1061 with P*mcr-1*_pWH13-*lacZ* transcriptional fusion at the chromosomal attB λ site	This work
FYJ850	DH5α (λ-*pir*) carrying pAH125-P*mcr-1* (pE15017)	This work
FYJ851	MC1061 with P*mcr-1*_pE15017-*lacZ* transcriptional fusion at the chromosomal attB λ site	This work
FYJ852	DH5α (λ-*pir*) carrying pAH125-*mcr-1* (pA31-12)	This work
FYJ853	MC1061 with P*mcr-1*_pA31-12-*lacZ* transcriptional fusion at the chromosomal attB λ site	This work
Plasmids		
pAH-P*fadD*	pAH125 carrying the *fadD* promoter region, Kan^R^	[31]
pAH-P*mcr-1*(pGD97)	pAH125 carrying the *mcr-1* promoter region from pGD97), Kan^R^	This work
pAH-P*mcr-1*(pWH13)	pAH125 carrying the *mcr-1* promoter region from pWH13, Kan^R^	This work
pAH-P*mcr-1*(pE15017)	pAH125 carrying the *mcr-1* promoter region from pE15017, Kan^R^	This work
pAH-P*mcr-1*(pA31-12)	pAH125 carrying the *mcr-1* promoter region from pA31-12, Kan^R^	This work

### Diversity in the *mcr-1*-harbouring plasmids

**Figure 2:**
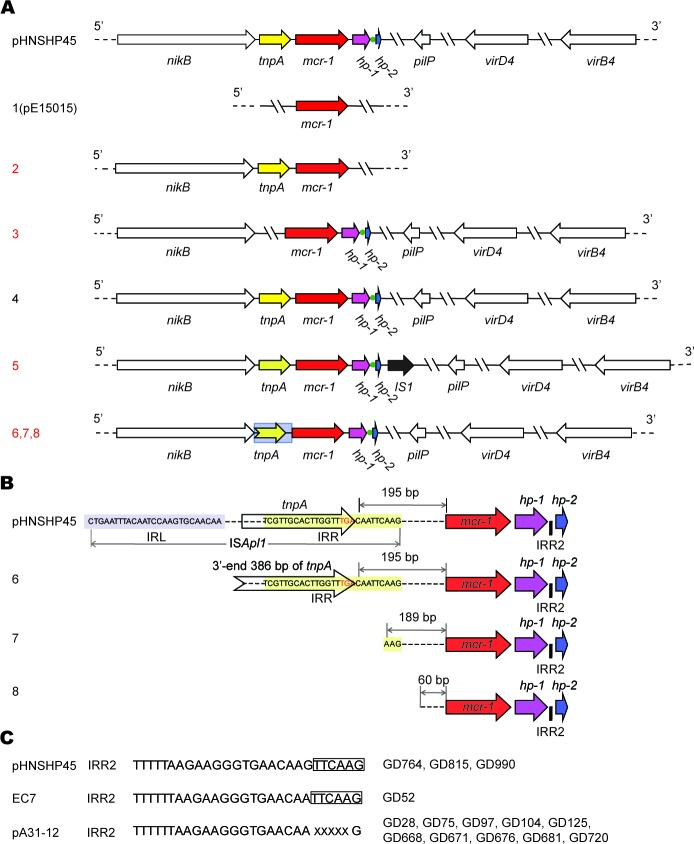
Genetic diversity in the *mcr-1*-harbouring plasmids from the swine gut microbiota **A.** Scheme of the eight types of the *mcr-1-*harbouring plasmids. The plasmid pHNSHP45 is a prototype with known genome sequence [8], whereas the other eight types of plasmids are revealed after PCR-based sequencing of the 89 representative *mcr-1*-containing plasmids collected from three pig farms in Guangdong province of China, in 2016. Arrows denote the known (and/or putative) genes/loci. The *mcr-1* gene is highlighted in red and 100% identical. The broken arrow marked in grey background denotes the partial sequence of *tnpA* gene at its 3′-end. The *tnpA* locus is PCR-negative in type 1 and 3. The green dot represents the IRR2 site. Apart from the type 1 and 2, all the other six types (3-8) are PCR-positive for the four genes (*nikB, pilP*, *virD4* and *virB4*), as well as a hypothetical protein (*hp*), adjacent to the 3′-end of *mcr-1*. Unlike the other types with an intact *tnpA* adjacent to the 5′-end of *mcr-1*, types 6, 7 and 8 feature with the truncated versions of *tnpA*. In type 1 like pE15015 we recently reported, only the *mcr-1* locus is PCR-positive. In type 2, two more loci (*nikB* and *tnpA*) can be detected in the PCR assays. In particular, an extra-insert IS1 transposase with 97% identity to the counterpart of *Acinetobacter baumannii* by BlastX (illustrated with a dark arrow) is closely present at the 3-end of *hp* in the type 5 of plasmid. In addition to the two known types (1 and 4) we reported very recently [9], we show six new plasmid types (2, 3, 5, 6, 7 and 8 labeled in red) in this study. **B.** Molecular insights into truncated versions of the *mcr-1*-containing IS*Apl1* mobile element. In the new plasmid types 6, 7 and 8, the *tnpA* transposase gene is variously featuring with truncated versions. Designations: IRL, Inverted Region Left; IRR, Inverted Region Right. **C.** Sequence feature of the IRR2 site. X denotes the variable nucleotide.

**Figure 3:**
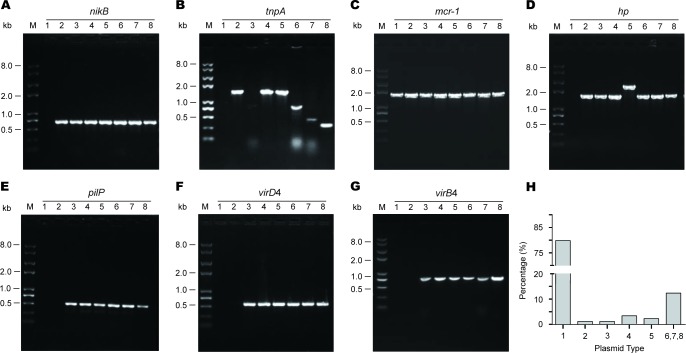
Genetic diversity of the *mcr-1*-harbouring plasmid backbones from the swine gut microbiota **A.** PCR-based discovery of *nikB*, the relaxosome protein. **B.** Molecular detection of *tnpA*, the transposase-encoding gene. **C.** PCR screen for the *mcr-1* gene. **D.** PCR detection of a putative protein (*hp*) gene that is supposed to be localized downstream of the *mcr-1* locus. **E.** PCR assay of the *pilP* gene encoding the type IV pilus biosynthesis protein. Molecular probing of a type IV secretion system-encoding genes *virD*4. **F.** and *virB*4. **G.** As we recently reported [9], we applied eight pairs of specific primers that are supposed to target the eight unique genes/loci localized on the paradigm *mcr-1*-harbouring plasmid pHNSHP45 [8]. According to the different profile of PCR amplicon, 8 types of genetic environment in total (numbered from 1, 2 … 8) are assigned to these *mcr-1*-positive plasmids from the swine gut bacteria. M denotes Trans 2K Plus II DNA Ladder (TRANSGEN BIOTECH, Beijing, China), and kb refers to kilo-base pair. **H.** Estimated distribution of *mcr-1*-carrying plasmids featuring with different genetic environment. Totally, 89 representative strains were tested here.

Given the fact the *mcr-1* gene can be surrounded with different genetic environment of diversified plasmid backbones in the case of human clinical *E. coli* isolates [9], we hypothesized that similar scenario could already be present in the animal isolates in the healthy swine populations (Figure 2). To address this hypothesis, hundreds of *mcr-1*-carrying *E. coli* isolates were subjected to extensive analyses using the multiplex-PCR assays coupled with Sanger sequencing (Figure 3). In terms of the paradigm *mcr-1*-harbouring plasmid pHNSHP45 with known genome sequence, seven pairs of specific primers (Table 2) that target seven interested genes (including *mcr-1* and *nikB*) were used in PCR assays to determine the various *mcr-1*-surrounding genetic environment (Figures 2 and 3). In addition to the *mcr-1* gene (Figure 3C), the remaining six genes corresponded to *nikB* (Figure 3A), *tnpA* (Figure 3B), *hp* (Figure 3D), *pilP* (Figure 3E), *virD4* (Figure 3F), and *virB4* (Figure 3G), respectively. The result of PCR assays coupled with Sanger sequencing revealed unexpected diversity of the *mcr-1*-harbouring plasmids (Figures 2 and 3), which is much more complicated than the scenarios seen in the human and animal *E. coli* isolates [9, 26].

**Table 2:** Primers used in this study

Primers	Primer sequence
*16S*-F	5'-AAA TTG AAG AGT TTG ATC ATG G-3′
*16S*-R	5'-GCT TCT TTA AGG TAA GGA GGT-3′
*mcr-1*-F	5'-ATG ATG CAG CAT ACT TCT GTG-3′
*mcr-1*-R	5'-TCA GCG GAT GAA TGC GGT G-3′
*nikB*-F	5'-GAT GAA CTT GAT CAT CGT GTT GT-3′
*nikB*-R	5'-GTA ATT CTG ACG AAA AAG AGG A-3′
*pilP*-F	5'-TTA AAG AAT AAG CTG GCG TTT C-3′
*pilP*-R	5'-ATG TTA AAA ATA ATT AAA CCA ACG-3′
*virD4*-F	5'-AAT GTC AAC ATG ATT GTT AC-3′
*virD4*-R	5'-GAA CAT AAC CCG GAC CTG AAA T-3′
*virB4*-F	5'-AAC TCT TTT TCA GTA AGC CCA AT-3′
*virB4*-R	5'-TTA ATG TTT GTT GTG GAT TAC AAC C-3′
*tnpA*-F	5'-GGT TTT CGG GCT TTT TAA GAG-3′
*tnpA*-R	5'-TAG CAC ATA GCG ATA CGA TG-3′
*hp*-F	5'-GAT AAG CAA ACT GGC ATC ACG-3′
*hp*-R	5'-GAA CCC TGT ATA TAG CCT GTC-3′
*adk*-F	5'-ATT CTG CTT GGC GCT CCG GG-3′
*adk*-R	5'-CCG TCA ACT TTC GCG TAT TT-3′
*fumC*-F	5'-TCA CAG GTC GCC AGC GCT TC-3′
*fumC*-R	5'-GTA CGC AGC GAA AAA GAT TC-3′
*gyrB*-F	5'-TCG GCG ACA CGG ATG ACG GC-3′
*gyrB*-R	5'-ATC AGG CCT TCA CGC GCA TC-3′
*icd*-F	5'-ATG GAA AGT AAA GTA GTT GTT CCG GCA CA-3′
*icd*-R	5'-GGA CGC AGC AGG ATC TGT T-3′
*mdh*-F	5'-ATG AAA GTC GCA GTC CTC GGC GCT GCT GGC GG-3′
*mdh*-R	5'-TTA ACG AAC TCC TGC CCC AGA GCG ATA TCT TTC TT-3′
*purA*-F	5'-CGC GCT GAT GAA AGA GAT GA-3′
*purA*-R	5'-CAT ACG GTA AGC CAC GCA GA-3′
*recA*-F	5'-CGC ATT CGC TTT ACC CTG ACC-3′
*recA*-R	5'-TCG TCG AAA TCT ACG GAC CGG A-3′
P*mcr-1*_A31-F(SalI)	5'-ACGC *GTCGAC* GAT ATT CAA CAG GTG ATC AAT AAA-3′
P*mcr-1*_A31-R(EcoRI)	5'-CCG *GAATTC* CAT GAG AAA CTA CTC AAA AAA TAA A-3′
P*mcr-1*_E15017-F(SalI)	5'-ACGC *GTCGAC* CTT GGA AAA CAA TTT GTC CAG G-3′
*lacZ*-R	5'-CAG TGA ATC CGT AAT CAT GGT C-3′

*The underlined sequences denote restriction enzyme sites

The integrated evidence accumulated here allowed us to temporarily divide them into eight groups (numbered with 1, 2, 3, …, 8) (Figure 2A). Type 1 plasmid is featuring with only PCR-positive for the *mcr-1* gene (Figures 2A and 3C). Besides the *mcr-1* gene, two more genes with expected size (*nikB* (Figure 3A) and *tnpA* (Figure 3B) are PCR-positive in Type 2 (Figure 2A). Unlike the type 4 plasmid whose PCR amplicons are identical to those of the paradigm *mcr-1*-containing plasmid pHNSHP45 (Figures 3A-3G) [8], Type 3 plasmid lacks the *tnpA* gene in front of the *mcr-1* locus (Figures 2A and 3B). In relative to the type 3 plasmid, Type 5 plasmid unexpectedly has a longer version of *hp* locus (Figure 3D) that was subsequently found to encode an extra-insert sequence (*IS1*) with 97% similarity to the counterpart in *Acinetobacter baumannii* (Figure 2A). In the following three types of plasmids (Types 6, 7 and 8), the *tnpA* loci exhibited with PCR amplicons of various lengths (Figure 3B).

### Variation in inverted repeat right (IRR)

Generally, the IS*Apl*1 insertion sequence is detected upstream of *mcr-1* by recognizing its own IRL (terminal inverted repeat left) and the closest downstream sequence that resembled its IRR (terminal inverted repeat right) (Figure 2B). The IRR2 seemed a flexible/imperfect IRR-like sequence (Figure 2C) [25]. Further genetic dissection defined that i) the terminal inverted repeat right (IRR, “TCGTTGCACTTGGTTTGACAATTCAAG”) remains 195 bp ahead of the *mcr-1* gene in Type 6 plasmid (Figure 2B); ii) the full sequence of IS*Apl1* is lost in the type 7 & 8 plasmids, remaining different relics with various DNA fragment left upstream of the *mcr-1* locus (189 bp for Type 7, and 60 bp for Type 8) (Figure 2B); iii) the site of IRR2 (Figure 2B) is consistently present in the inter-genic region between *hp-1* and *hp-2* (Figure 2C). IRR2 of GD52 other than GD764, GD815, and GD990 is identical to that pHNSHP45 (TTTTTAAGAAGGGTGAACAAGTTCAAG)Intriguingly, the IRR2 (TTTTTTAAGAAGGGTGAACAAXXXXXG) of pA31-12 [25] was seen in ten strains/plasmids like GD28, GD75, GD97, etc. (Figure 2C).

Extensive analysis of 89 *mcr-1*-containing sequences suggested that 80% of plasmids belonged to Type 1, and the plasmid of Type 6/7/8 (15% in total) is next to Type 1 (Figure 3H). Unlike the plasmids of the prevalent type 1, the plasmids of Type 2 (or 3) are pretty rare (1-2%, Figure 3H). Given the fact that only Type 1 and 3 plasmids have ever been observed in our earlier investigation [9, 12, 13], our findings here extended significantly the proposal that the *mcr-1* gene is carried by plasmids with diversified genetic environments.

### Complexity in sequence types of the *mcr-1*-positive *E. coli* isolates

To test the genetic heterogeneity amongst the *mcr-1*-positive *E. coli* isolates, we thus performed the analyses of multi-locus sequence typing (MLST) using 15 representative *E. coli* isolates [23]. The MLST-based sequence typing showed that they comprise 10 different sequence types (STs) including ST98, ST10, ST20, ST218, ST165, ST641, ST93, ST1286, ST4656, ST3546, and a novel ST (*n* = 2) (Table 3). The novel ST (allelic profile: 10-11-5-10-8-236-2) is assigned to two *E. coli* isolates GD97 and GD676. Most of the STs we determined, were not previously related to the *mcr-1* gene. But the ST10 *E. coli* in Belgium was ever found to carry ESBL-producing plasmids and associated with human infection [27]. By contrast, we recently observed co-production of MCR-1 and ESBL in the epidemic strain of ST648 *E. coli* [23]. Obviously, the *mcr-1-*positive *E. coli* isolates from the swine populations exhibited appreciably genetic heterogeneity in terms of diversified STs (Table 3).

**Table 3:** Sequence typing of the mcr-1-positive *E. coli* strains

Strains	Alleles							ST	ST Complex
	*adk*	*fumC*	*gyrB*	*icd*	*mdh*	*purA*	*recA*		
MG1655	10	11	4	39	8	8	2	ST98	ST10 Cplx
GD28	10	11	4	8	8	8	2	ST10	ST10 Cplx
GD428	10	11	4	8	8	8	2	ST10	ST10 Cplx
GD703	10	11	4	8	8	8	2	ST10	ST10 Cplx
GD75	6	4	3	18	7	7	6	ST20	ST20 Cplx
GD104	6	4	3	18	7	7	6	ST20	ST20 Cplx
GD125	6	4	3	18	7	7	6	ST20	ST20 Cplx
GD66	10	11	4	12	8	8	2	ST218	ST10 Cplx
GD668	10	27	5	10	12	8	2	ST165	ST165 Cplx
GD788	9	6	33	131	24	8	7	ST641	ST86 Cplx
GD811	6	11	4	10	7	8	6	ST93	ST168 Cplx
GD720	10	174	4	8	8	8	2	ST1286	/
GD815	6	11	4	8	8	18	2	ST4656	/
GD819	10	11	57	8	8	8	20	ST3546	/
GD97	10	11	5	10	8	N/A	2	New ST	/
GD676	10	11	5	10	8	N/A	2	New ST	/

### The *mcr-1* promoter

**Figure 4:**
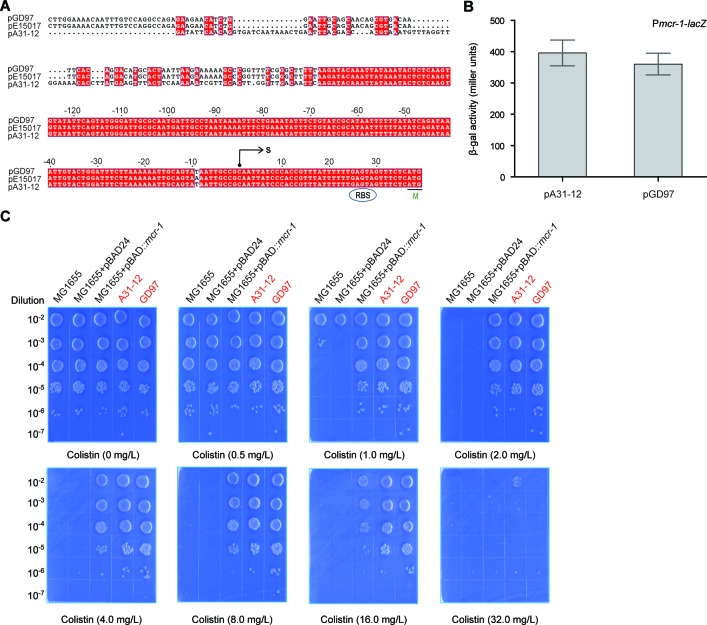
The *mcr-1* promoter activity determines the level of the *E. coli* resistance to colistin **A.** Bioinformatics analyses of the *mcr-1* promoter. The putative *mcr-1* promoter sequences were derived from the two Guangdong's *mcr-1*-positive plasmids (pA31-12 [25] and pGD97, a representative of plasmids we report here). The nucleotide sequences were aligned with ClustalW2 (http://www.ebi.ac.uk/Tools/clustalw2/index.html), and the resultant output was generated with ESPript 2.2 (http://espript.ibcp.fr/ESPript/cgi-bin/ESPript.cgi) [34, 35]. Identical residues are white letters with red background, similar residues are black letters, and gaps are denoted with dots. Designations: S, transcription start site; M, methionine and translation initiation site; RBS, ribosome binding site. **B.** Measurement of β-gal activity driven by the *mcr-1* promoter. To measure bacterial β-gal activity, mid-log phase cultures of *E. coli* MC1061 with the P*mcr-1-lacZ* transcriptional fusion on chromosome were collected by spinning and suspended in Z-buffer [36]. The data was recorded in triplicate in three independent experiments. The two strains used here include FYJ853 (P*mcr-1_*pA31-12-*lacZ* for the *mcr-1* promoter of A31-12 strain) and FYJ 851 (P*mcr-1_*pGD97-*lacZ* for the *mcr-1* promoter of GD97 strain). Note: the *mcr-1* promoter sequences (~300 bp region upstream the transcription start site C) are identical. **C.** Visualization of the colistin resistance of *E. coli* conferred by the *mcr-1*-harbouring plasmids. The *E. coli* strain MG1655 with/without the pBAD24 vector acts as the negative control, whereas the MG1655 with the arabinose-driven MCR-1 expression refers to the positive control. The remaining two (A31-12 and GD97) are clinical *mcr-1*-positive isolates (highlighted in red). The minimum inhibitory concentration (MIC) of the colistin was determined with the method of plating in series of dilution. Briefly, the mid-log phase cultures (OD600 = 0.8) in serial dilution were spotted on LBA plates with different level of colistin (0, 0.5, 1.0, 2.0, 4.0, 8.0, 16.0 and 32.0 mg/L) and 0.2% arabinose. The LBA plates were maintained overnight at 37°C.

The *mcr-1* promoter regions from the two plasmids (pA31-12 and pGD97) were compared using multiple sequence alignment, indicating that it is not less conserved at 5′-end than that at 3′-end (Figure 4A). Prokaryotic promoter analysis by the Neutral Network Program of Promoter Prediction (http://www.fruitfly.org/seq_tools/promoter.html) suggested that the transcription start site of the *mcr-1* gene is C at the position of 36 bp upstream of the translation initiation site “ATG” (Figure 4A), which is 1 bp upstream in comparison with the transcription start site “A' revealed by Poirel *et al*. [28] with 5′-RACE. The minor difference might be due to mapping of the truncated version of *mcr-1* transcript by 5′-RACE. To evaluate the promoter activity of the *mcr-1* gene, the *mcr-1* promoter regions (303 bp) of the two plasmids (pA31-12 and pGD97) were fused to the promoter-less *lacZ* gene, giving P*mcr-1-lacZ* transcriptional fusions. Subsequent LacZ analyses showed that β-gal activity driven by the *mcr-1* promoters of both pGD97 and pA31-12 is ~400 miller units (Figure 4B). It suggested that both pGD97 and pA31-12 has a *mcr-1* promoter with comparable medium strength. Intriguingly, this speculation was proved by the fact the two *mcr-1*-positive strains (GD97 and A31-12) exhibited a similar level of colistin resistance to colistin (~16 mg/L) (Figure 4C). Given the fact that colistin resistance by the arabinose-induced expression of MCR-1 is similar to that of GD97 and A31-12, we therefore concluded that *mcr-1* promoter is comparable to the arabinose-inducible promoter of pBAD24.

## CONCLUSIONS

Colistin is a last defense against lethal infections by Gram-negative pathogens with multiple drug resistance. The MCR-1 mediates the transferable resistance to colistin, raising an old, but newly-emerging threat to public health [10]. In the past six months since the first discovery of the *mcr-1* colistin resistance gene in Southern China [8], the literatures regarding to the *mcr-1*/colistin resistance are increasingly-accumulated (http://www.ncbi.nlm.nih.gov/pubmed/?term=mcr+1+colistin). Among them, most of studies concentrated on molecular epidemiology of the *mcr-1* transmission/dissemination. In particular, unexpected complexity in the multi-drug resistance was assigned to the *mcr-1*-positive enteric bacteria [14, 23]. More seriously, the MCR-1 was found to co-localize with two types of notorious drug resistant genes (ESBL [20, 23, 26] and NDM-1 (and/or its mutants) [29, 30]. We are first to report the diversified *mcr-1*-harbouring plasmids from clinical *E. coli* isolates of diarrhea patients in China [9]. The similar scenarios were also seen in the *E. coli* isolates from the swine populations [12].

In this study, we screened over 1000 pieces of *E. coli* samples (from Guangdong Province, China) for the presence of the *mcr-1* gene (Figure 1). It allowed us to gain insights into genetic environment of the *mcr-1*-bearing plasmids. In addition to the two known plasmid types (types 1 and 3), we observed four more kinds of plasmid types (Figure 2), suggesting the unprecedented complexity in the *mcr-1*-carrying plasmids. Also, the variation is present in the IRR2 motif. It is not surprise for us to notice nearly 10 different sequence types can be assigned to these *mcr-1*-positive *E. coli* isolates. To the best of our knowledge, it might represent an example of complicated genetic diversity in both *mcr-1*-carrying plasmids and *E. coli* hosts originating from the healthy swine populations in China. Because that the IRR sites are involved in the transposon-like transferable events of the *mcr-1* gene, the various versions of IRR2 might imply difference/diversity among the MCR-1 transfer. Given the fact that the high prevalence and complexity of the MCR-1 colistin resistance in the healthy swine populations in China, it is reasonable that enhanced surveillance efforts is warranted to monitor and/or control the spread of the *mcr-1* resistance gene, *esp.* the possible dissemination of food chain. Additionally, we are first to showe *in vitro* evidence that the level of colistin resistance in various clinical strains is determined by the promoter activity of the *mcr-1* gene (Figure 4B).

In summary, it seems likely that complex dissemination of the diversified *mcr-1*-harbouring plasmids occurs amongst the various ST *E. coli* inhabiting the healthy swine populations, in Southern China. In particular, our findings highlighted the urgent need to reconsider the efficacy (safety) of colistin in the veterinary use, and formulate a comprehensive strategy to fight against the diversified plasmid-mediated *mcr-1* colistin resistance in pan-drug-resistant Gram-negative bacteria.

## MATERIALS AND METHODS

### Bacterial isolations and identification

All the bacterial strains were *E. coli* derivatives (Table 1). Fecal samples were routinely collected from three different pig farms in Guangdong province, China, in 2016. The nasal fluid and feces of pigs were sampled. MacConkey solid agar plates were applied to isolate the Enterobacterial species. The resultant bacteria were subjected to colony PCR assays for the presence of the *mcr-1* gene (Table 2).

### DNA manipulations

To address the genetic context surrounding the *mcr-1* gene, all of the *mcr-1*-positive *E. coli* isolates were subjected to the multiplex-PCR with six pairs of specific primers (Table 2) [9]. The resultant PCR products were determined with Sanger sequencing. To probe the genetic heterogeneity of the *mcr-1*-positive isolates, MLST was carried out as we described [23]. Seven house-keeping genes examined here correspond to *adk*, *fumC*, *gyrB*, *icd*, *mdh*, *purA* and *recA*, respectively. The relevant seven pairs of primers were available in the server of MLST (http://mlst.warwick.ac.uk/mlst/dbs/Ecoli).

### Plasmids and genetic manipulations

The *mcr-1* promoters from the following plasmids (pA31-12, pE15017 and pGD97) were amplified with PCR and directly cloned into pAH125, giving the *lacZ* transcriptional fusions, P*mcr-1-lacZ* (Table 1). As we described earlier [31], the resultant recombinant plasmids like pAH-P*mcr-1*(pA31-12), were transformed into MC1061, and screened on Luria-Bertani Agar (LBA) plates containing kanamycin and x-gal to give the chromosomal *mcr-1* transcriptional *lacZ* fusion strains like FYJ853 (Table 1).

### β-Galactosidase assays

The strains of *E. coli* carrying the chromosome P*mcr-1-lacZ* transcriptional fusion (e.g., FYJ853) were grown in LB media, and mid-log phase cultures were collected assayed for β-galactosidase activity following lysis with SDS (sodium dodecylsulfate)-chloroform [31, 32]. The data were recorded in triplicate from three independent experiments.

### Determination of the colistin resistance/tolerance

Antibiotic susceptibility experiments were conducted by the agar dilution method [33] recommended by the Clinical and Laboratory Standards Institute guidelines (CLSI M100-S25). The EUCAST breakpoint for *Enterobacteriaceae* was applied for colistin resistance (European Committee on Antimicrobial Susceptibility Testing 2015). Here, strain FYJ795 refers to the positive control, whereas the two strains (MG1655 and FYJ796) are the negative controls. All strains including A31-12 and GD97 were collected in Mid-log-phase, then bacterial cultures in a dilution series were spotted on LBA plates supplemented with various levels of colistin (0, 0.5, 1.0, 2.0, 4.0, 8.0, 16.0, and 32.0 mg/liter) and maintained at 37°C overnight. When necessary, 0.2% arabinose was added into the LBA plates for induction of MCR-1 expression [9].
